# Supplementary material for: Innate immune evasion revealed in a colorectal zebrafish xenograft model
Source: Nat Commun. 2021 Feb 19;12:1156. doi: 10.1038/s41467-021-21421-y (PMC7895829; doi:10.1038/s41467-021-21421-y)
Supplement: Supplementary file 1 — Supplementary Information [file 41467_2021_21421_MOESM1_ESM.pdf]

Supplementary Information

Innate immune evasion revealed in a colorectal zebrafish xenograft model

(Póvoa et al.)

Supplementary Figure 1

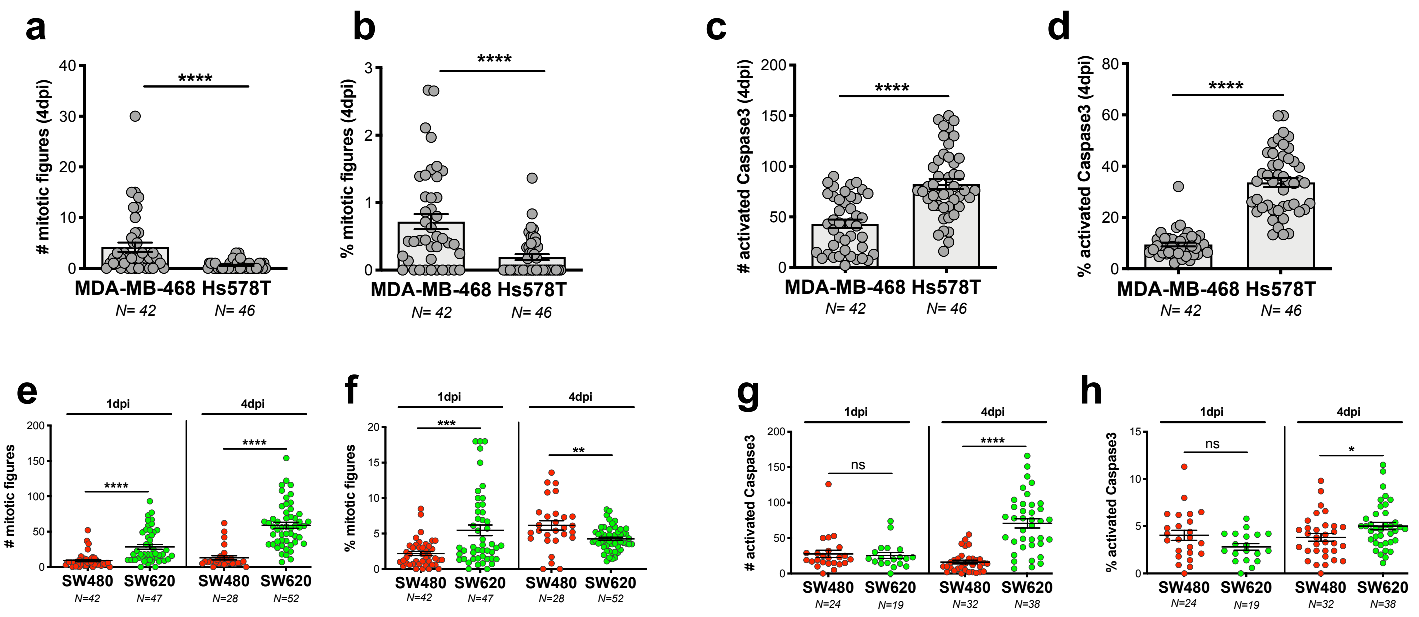

Supplementary Figure 1 | Engraftment does not seem to correlate with proliferation potential or cell death. a-d. Quantification of proliferation and apoptosis in triple negative breast cancer cell lines MDA-MB-468 and Hs578T. Mitotic figures absolute numbers (**a**) and % (**b**) at 4dpi (unpaired two-sided Mann Whitney test ****P<0.0001). Absolute numbers **(c)** and % of activated Caspase3 (**d**) at 4dpi (unpaired two-sided Mann Whitney test ****P<0.0001). **a-d.** Error bars indicate mean ± SEM from 3 independent experiments. **e-h.** Quantification of proliferation and apoptosis in CRC cell lines SW480 and SW620. Mitotic figures absolute numbers (**e**) and % (**f**) at 1dpi and 4dpi (unpaired two-sided Mann Whitney test ****P <0.0001, ***P=0.0007, **P=0,002). Absolute numbers (**g**) and % (**h**) of activated Caspase3 at 1dpi and 4dpi (unpaired two-sided Mann Whitney test ns=0.94, ****P< 0.0001, ns=0.11, *P=0.03). **e-h**. Error bars indicate mean ± SEM from 4 independent experiments. Each dot represents a xenograft. N is depicted in the charts.

Supplementary Figure 2

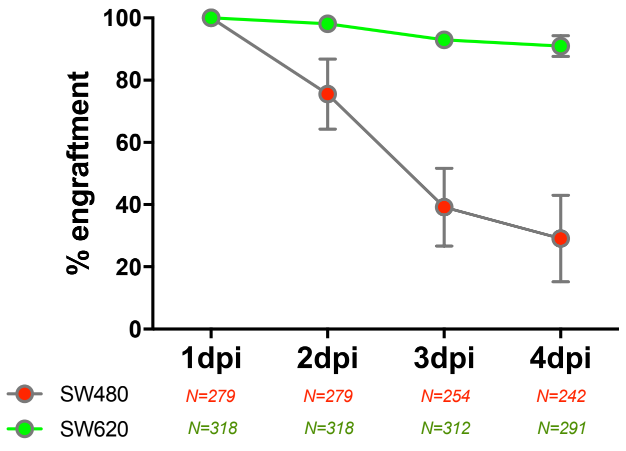

Supplementary Figure 2 | SW480 tumor cell clearance through time in the zebrafish host. Engraftment rate in SW480 and SW620 zebrafish xenografts were quantified at the indicated time post injection. Data are average of 3 independent experiments, total number of analyzed xenografts are depicted in the chart (SW480 in red and SW620 in green). Error bars indicate mean ± SEM.

Supplementary Figure 3

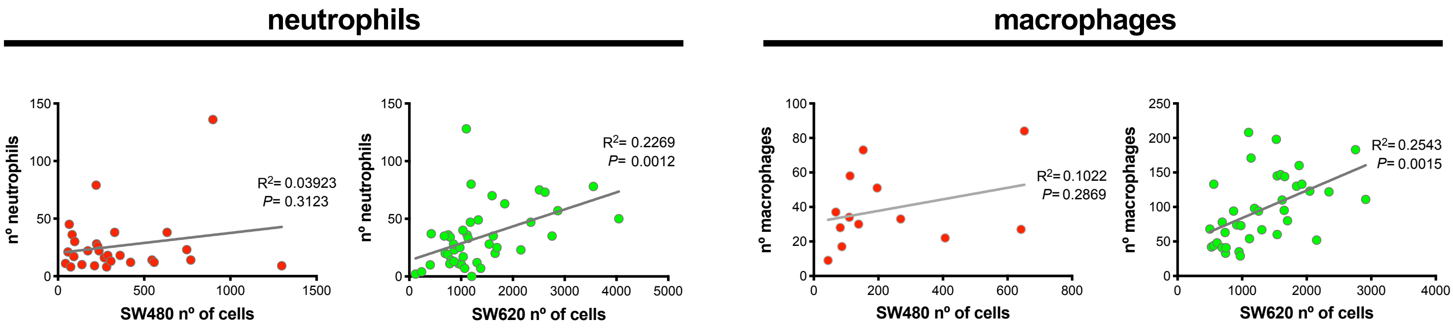

Supplementary Figure 3 | Correlation between tumor cell number and respective innate immune cell infiltrate. The correlation between neutrophils and macrophages with the number of SW480 and SW620 tumor cells was evaluated using Spearman's correlation test and linear regression analysis. R^2 and *P* values indicated in the charts. SW480 vs neutrophils N=28 xenografts, SW620 vs neutrophils N=43 xenografts, SW480 vs macrophages N=13 xenografts, SW620 vs macrophages N=37 xenografts.

Supplementary Figure 4

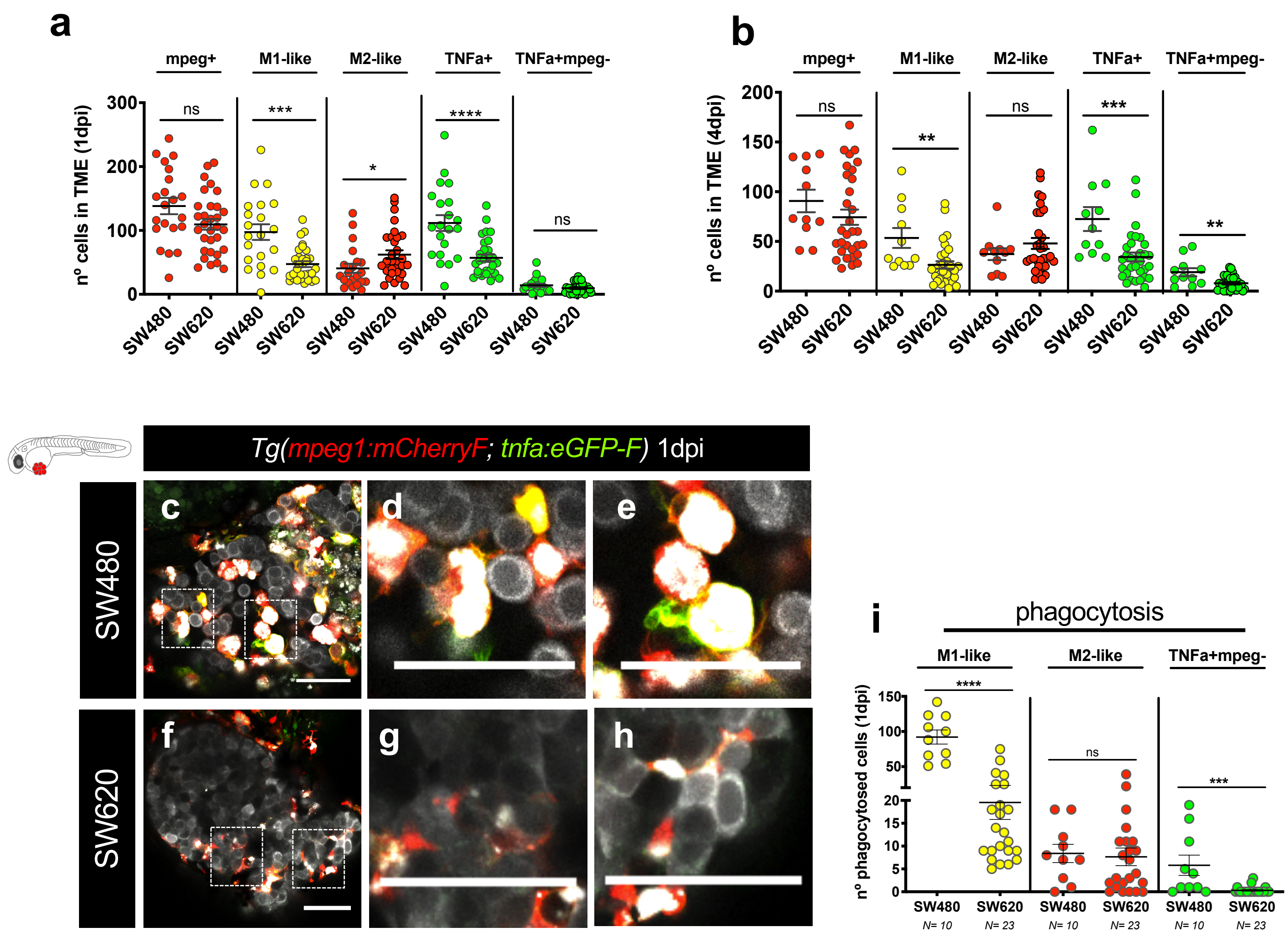

Supplementary Figure 4 | SW480_regressor tumors present more inflammatory and phagocytic macrophages than SW620_progressors. a-b. Quantification of absolute numbers of overall macrophages (mpeg+), M1-like macrophages (mpeg+;TNFa+), M2-like macrophages (mpeg+;TNFa-), overall TNFa+ cells and TNFa+mpeg- cells at 1dpi (**a**, ns=0.068, ***P=0.0003, *P=0.012,****P<0.0001, ns=0.1) and 4dpi (**b**, ns=0.17, **P=0.001, ns=0.52, ***P<0.0009, **P=0.006) of SW480 and SW620 xenografts. SW480_1dpi N=21 xenografts, SW620_1dpi N=31 xenografts, SW480_4dpi N=11 xenografts, SW620_4dpi N=31 xenografts. **c-h**. Representative confocal images of SW480 (**c-e**) and SW620 (**f-h**) tumor cells (in white) and macrophages (in red), showing macrophage-tumor close interaction using *Tg(mpeg1:mCherryF; tnfa:eGFP-F)*. **d-e** and **g-h.** Higher magnification of phagocyted tumor cells of **c** and **f**, respectively indicated by the dashed lines. **i.** Absolute quantification of phagocytosed tumor cells by M1, M2-like macrophage and TNFa+mpeg- inflammatory cells at 1dpi (****P<0.0001, ns=0.50, ***P=0.0002). Each dot represents one xenograft. Error bars indicate mean ± SEM from 3 independent experiments. All data was analyzed using unpaired two-sided Mann Whitney test. Scale bars: 50um.

Supplementary Figure 5

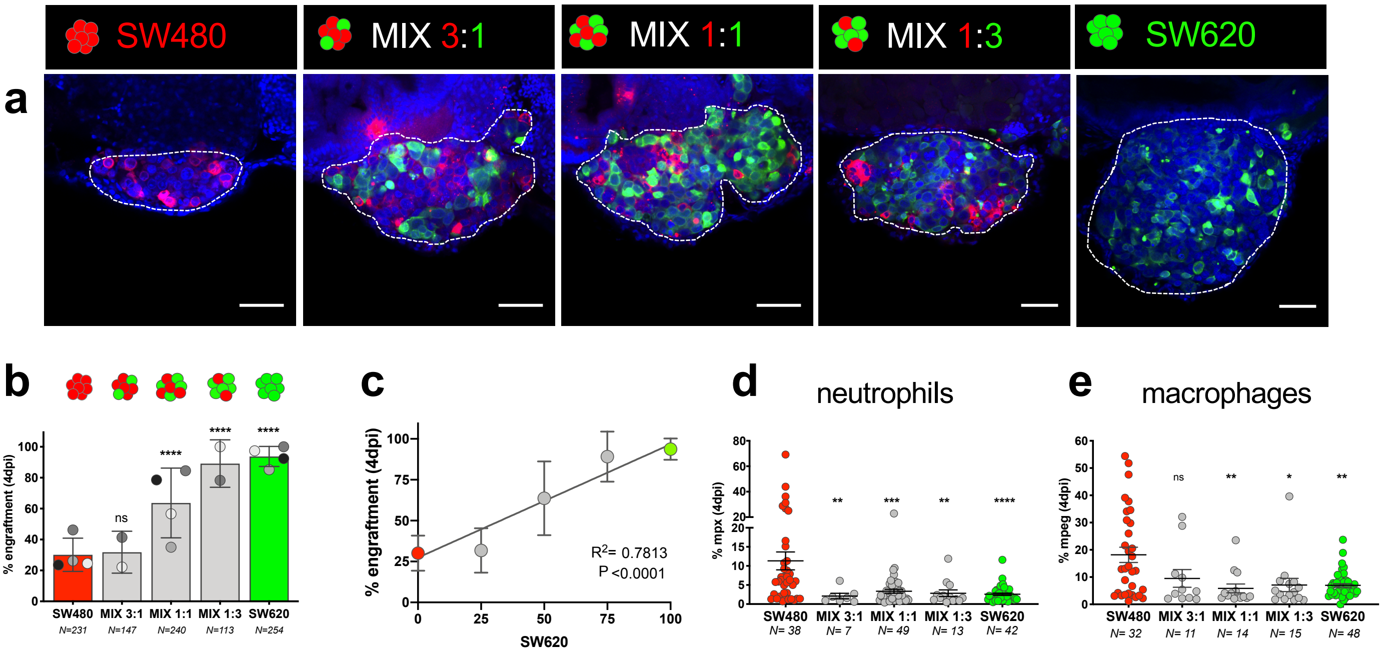

Supplementary Figure 5 | Engraftment of MIX xenografts correlates with the number of SW620 cells present in the tumor. a. Representative confocal images of SW480, SW620 and MIX zebrafish tumors at 4dpi. Mixtures were 75%, 50% and 25% SW480 (red) mixed with 25%, 50% and 75% cells of SW620 (green), respectively. **b.** Quantification of engraftment at 4dpi, Fisher exact test ns=0.73, ****P<0.0001. Error bars indicate mean ± S.D. Each dot represents one independent experiment, and each set of independent experiments is represented in a different gray color. **c.** Linear regression analysis and Spearman's test of number of SW620 cells against tumor engraftment. Each dot represents the engraftment mean from f. **d.** Quantification of neutrophils within SW480, SW620 and MIX tumors at 4dpi, unpaired two-sided Mann Whitney test 480 vs MIX 3:1 **P=0.0066, Cohen's D g=0,67; ***P=0.0002, Cohen's D g=0,79; 480 vs MIX 1:3 **P=0.0029, Cohen's D g=0,66;****P<0.0001, Cohen's D g=0,86. **e.** Quantification of macrophages in SW480, SW620 and MIX tumors at 4dpi, unpaired two-sided Mann Whitney test ns=0.0586, Cohen's D g=0,57; 480 vs MIX 1:1 **P=0.0025, Cohen's D g=0,88; *P=0.0107, Cohen's D g=0,77; 480 vs 620 **P=0.0089, Cohen's D g=1,05. Each dot represents one xenograft. **d-e**. Error bars indicate mean ± SEM (from 3 independent experiments). Dashed lines encircle tumor areas. Nuclei are stained with DAPI. N is depicted in the chart. Scale bars: 50μm.

Supplementary Figure 6

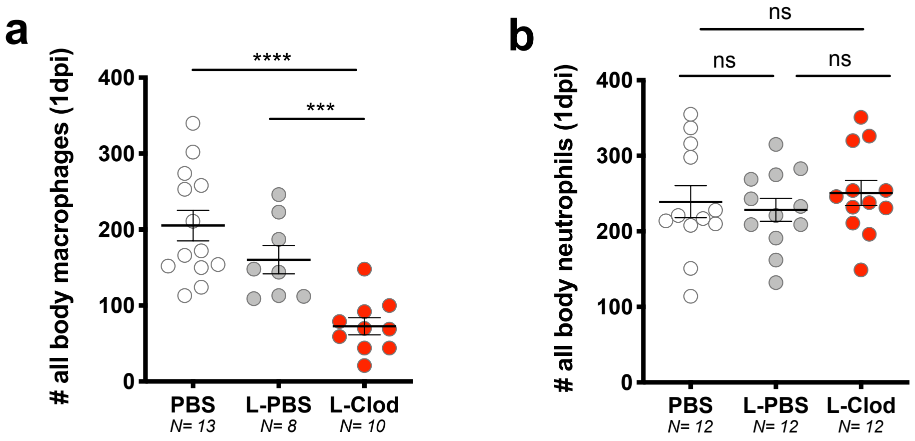

Supplementary Figure 6 | Clodronate treatment reduces the number of macrophage but not neutrophils. Number of total macrophages (**a**) and neutrophils (**b**) at 1dpi in SW480 zebrafish xenografts treated with PBS, L-PBS and L-clodronate. PBS vs L-Clod ****P<0,0001, Cohen's D g=2,1; L-PBS vs L-Clod ***P=0,0007, Cohen's D g=1,9. N is depicted in the chart. Each dot represents one xenograft. Error bars indicate mean ± SEM (from 1 independent experiment). Macrophages and neutrophils were quantified based on the *Tg(mpeg1:mCherry-F)* and *Tg(mpx:eGFP)*, respectively. Data was analyzed using unpaired two-sided t test.

Supplementary Figure 7

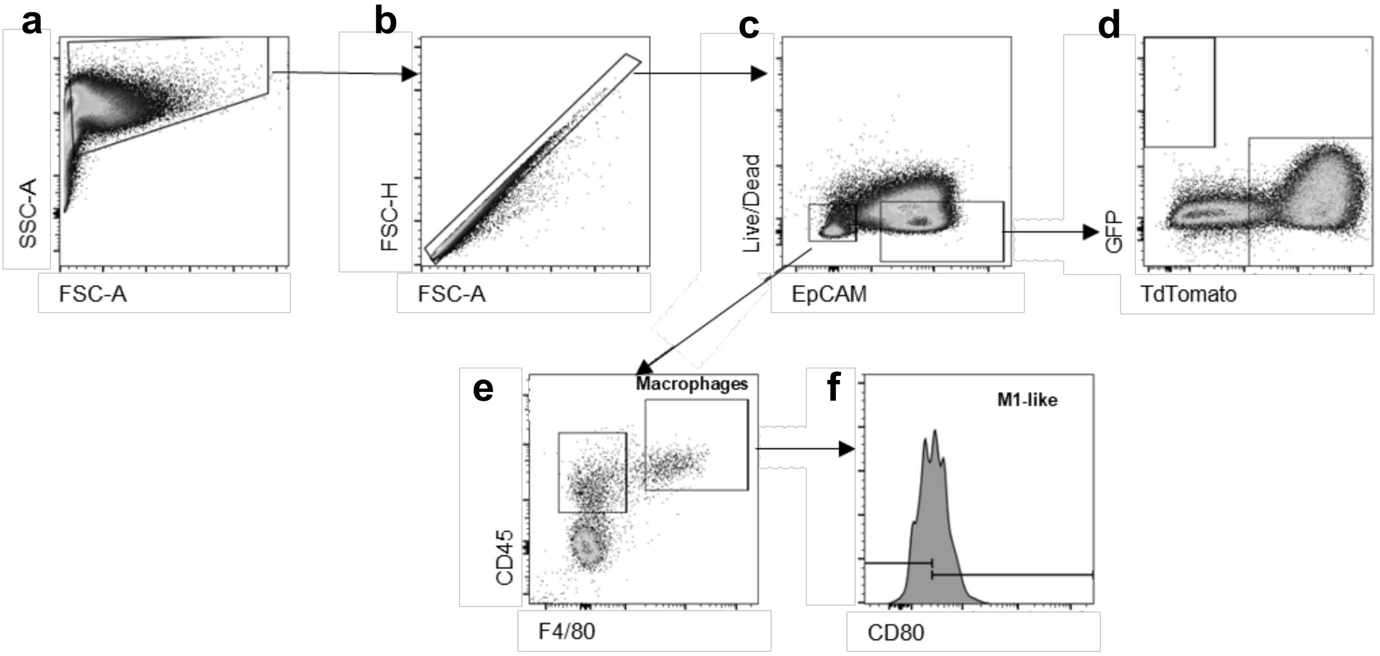

Supplementary Figure 7 | Gating strategy for flow cytometry analysis
Gating strategy to determine the percentages of Human cells (LIVE/DEAD-EpCAM+), SW620 cells (LIVE/DEAD-EpCAM+TdTomato+GFP-), SW480 cells (LIVE/DEAD-EpCAM+TdTomato-GFP+), Mouse cells (LIVE/DEAD-EpCAM-), Macrophages (LIVE/DEAD-EpCAM-CD45+F4/80+) and anti-tumoral M1-like macrophages (LIVE/DEAD-EpCAM-CD45+F4/80+CD80+) in mouse xenografts described on Fig. 6. Single cell suspension of tumor xenografts were gated as shown in **a** to eliminate debris and doublets were discarded as shown in **b.** Live cells were gated by exclusion of Live/Dead-Aqua positive cells as shown in **c.** Live human cells were gated as Live/Dead-Aqua negative and EpCAM-APC positive shown in **c** and live mouse cells were gated as Live/Dead-Aqua negative and EpCAM-APC negative **c**. SW480 cells were gated as GFP-FITC positive and PE-TdTomato negative as shown in **d.** SW620 cells were gated as GFP-FITC negative and PE-TdTomato positive**.** Macrophages were selected by double expression of CD45-APCCy7 and F4/80-PECy7 as shown in **e** and were gated by exclusion of human cells (shown in **c**) and through the *Live Mouse* gate **e.** M1-like macrophages were selected by expression of CD80-FITC within the *Macrophages* gate as shown in **f.**

Supplementary Figure 8

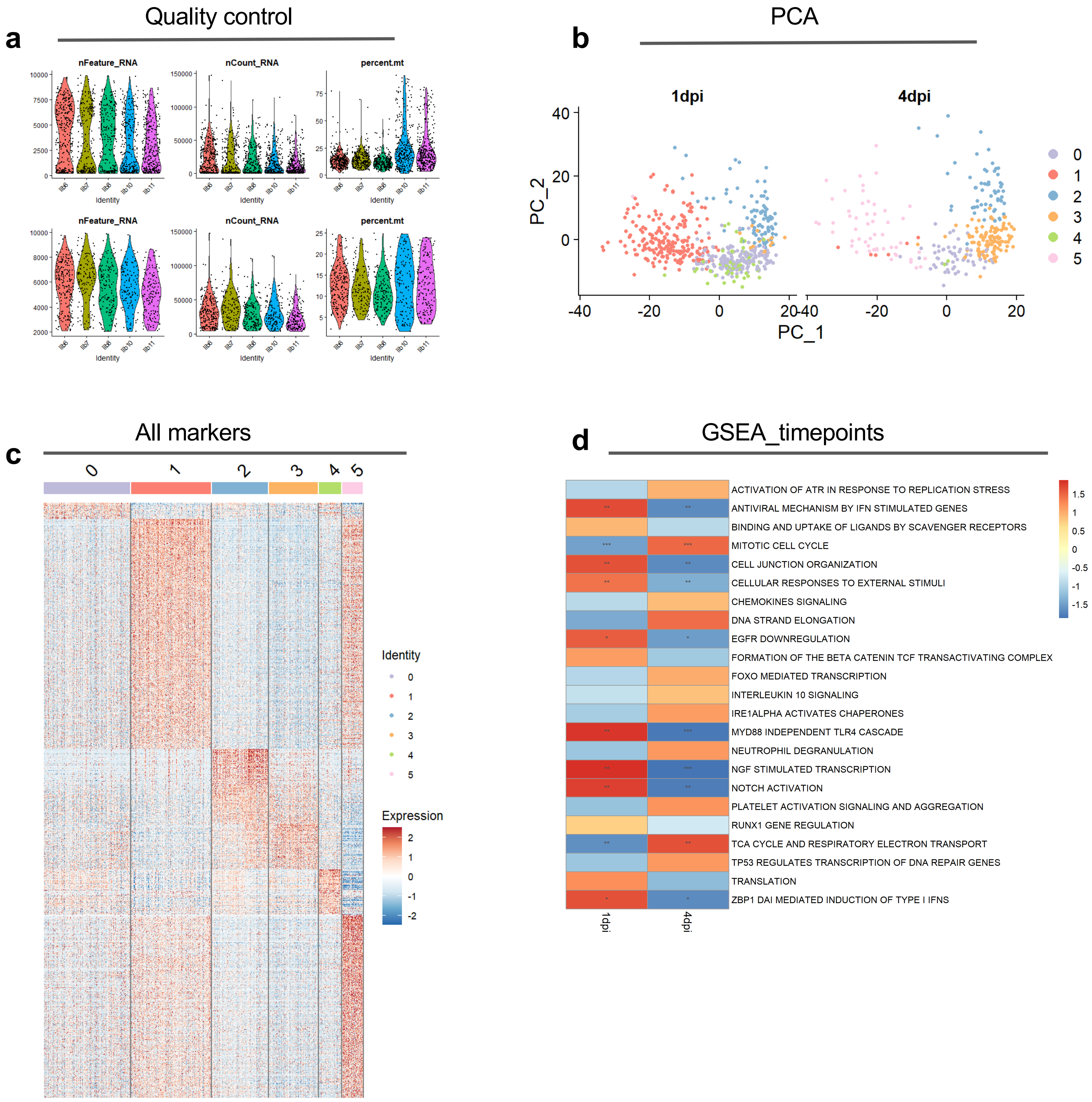

Supplementary Figure 8 | a. Quality control plots of the five sequenced single-cell libraries (lib6, 7 and 8 for 1dpi and lib10 and 11 for 4dpi). On the top three panels, we see the data before filtering representing total number of genes detected by barcode, the number of total unique reads by barcode and the number of unique reads associated with mitochondrial genes. The bottom three panels represent the same data after filtering cells with less than 2000 genes detected and with more than 25% of reads associated with mitochondrial genes; **b.** Principal Component Analysis (PCA) plot, colored by cell cluster and divided by time point. **c.** Heatmap of normalized expression values of all significant marker genes (adjusted p-value < 0.05), where colors represent expression values scaled by row (Z-scores). **d.** Heatmap representation of Normalized Enrichment Scores (NES) of representative pathways (from Figure 8d) in Gene Set Enrichment Analysis (GSEA), comparing the gene expression of cells at 1dpi with cells at 4dpi. Red colors mean that genes in that pathway tend to be more expressed in that group, while blue means that genes tend to be less expressed. Significant NES values are marked with asterisk (Fisher exact test *: adjusted p-value < 0.05; **: adjusted p-value < 0.01; ***: adjusted p-val <0.001).

Supplementary Figure 9

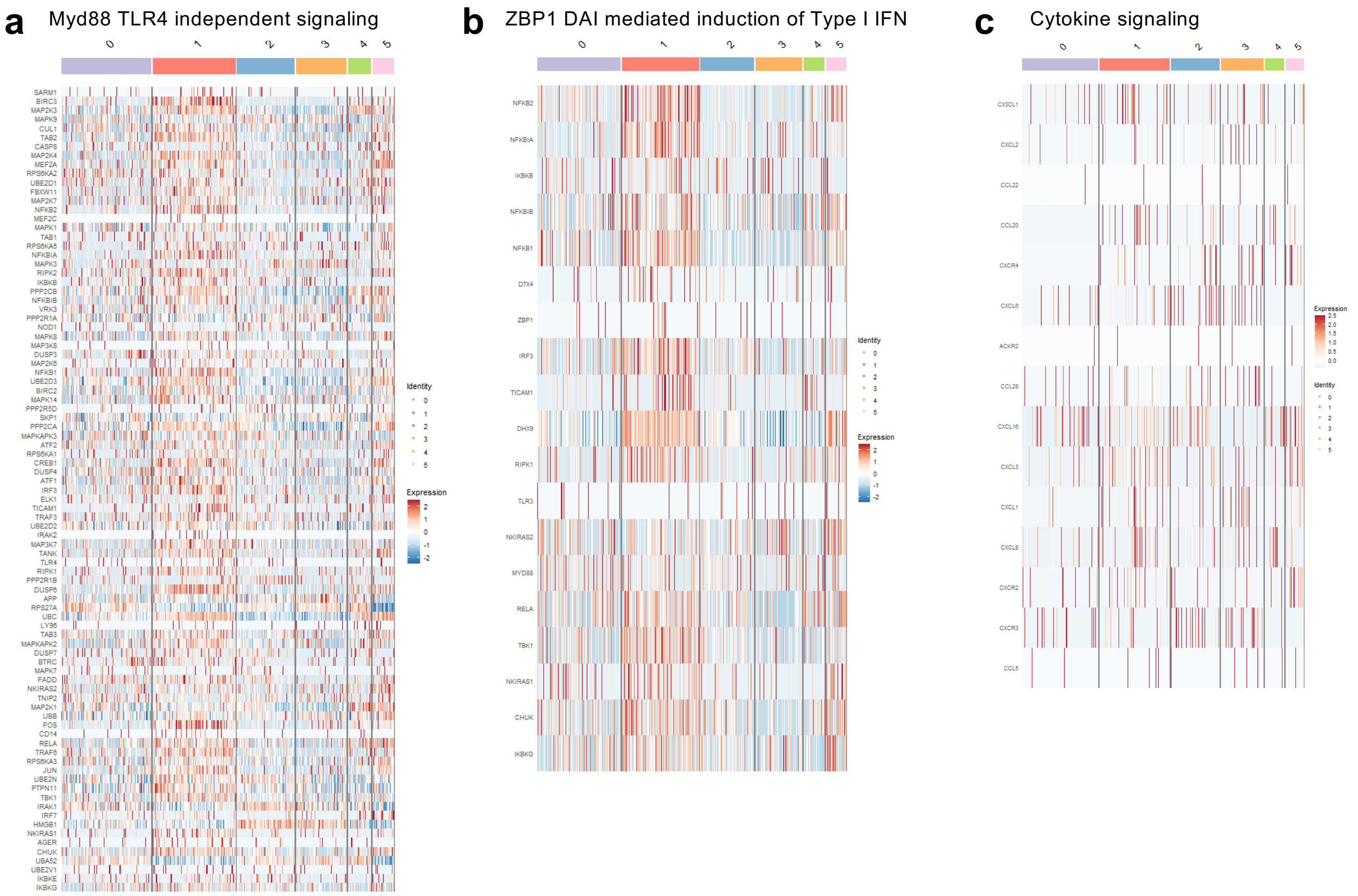

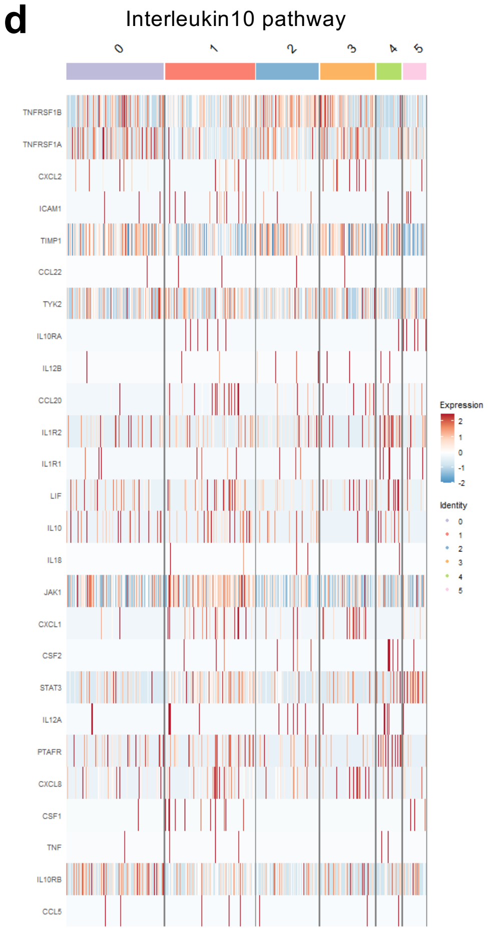

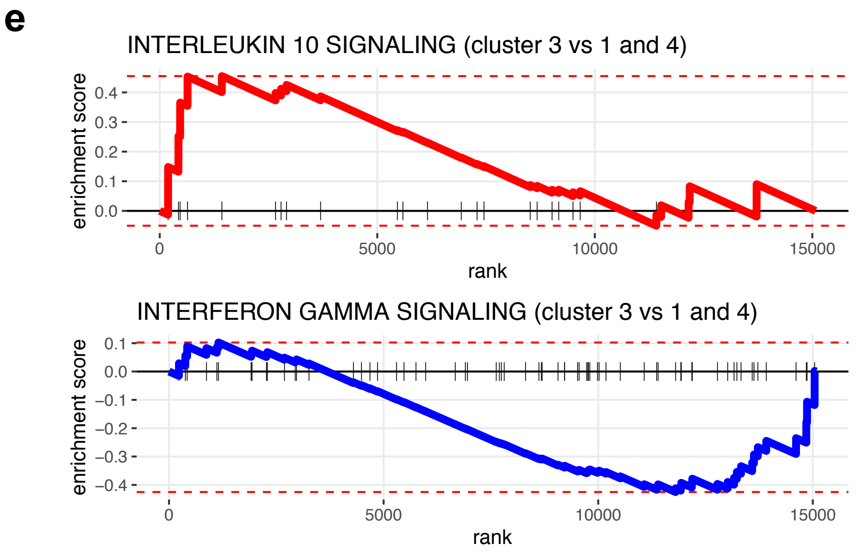

Supplementary Figure 9 | a-d. Heatmap of normalized expression values of genes belonging to significantly enriched pathways from the Molecular Signatures Database (MSigDB), namely **a.** Myd88 TLR4 independent signaling: (cluster0: NES=-1,6, padj=0,003; cluster1: NES=1,6, padj= 0,0008; cluster2: NES=-1,7, padj= 0,0006; cluster3: NES=-1,5, padj=0,006); **b.** ZBP1 DAI mediated induction of type I interferon: (cluster0: NES0 -1,7, padj=0,01; cluster1: NES= 1,7, padj=0,003; cluster2: NES=-1,6, padj=0,0075; cluster3: NES=-1,5, padj= 0,016); **c.** chemokine signaling: (cluster2: NES=-1,7, padj= 0,001; cluster3: NES= 1,9, padj=0,002); **d.** IL10 pathway: (cluster3: NES=1,8, padj= 0,007). Colors represent expression values scaled by row (Z-scores). **e.** GSEA of IL 10 signaling (NES=1.25,, pval=0.27) and interferon gama signaling (NES=-1.1, pval=0.3) signatures comparing cluster 3 (expands) vs 1 and 4 (that reduce frequency). All statistical analysis were performed using Fisher exact test.

Supplementary Figure 10

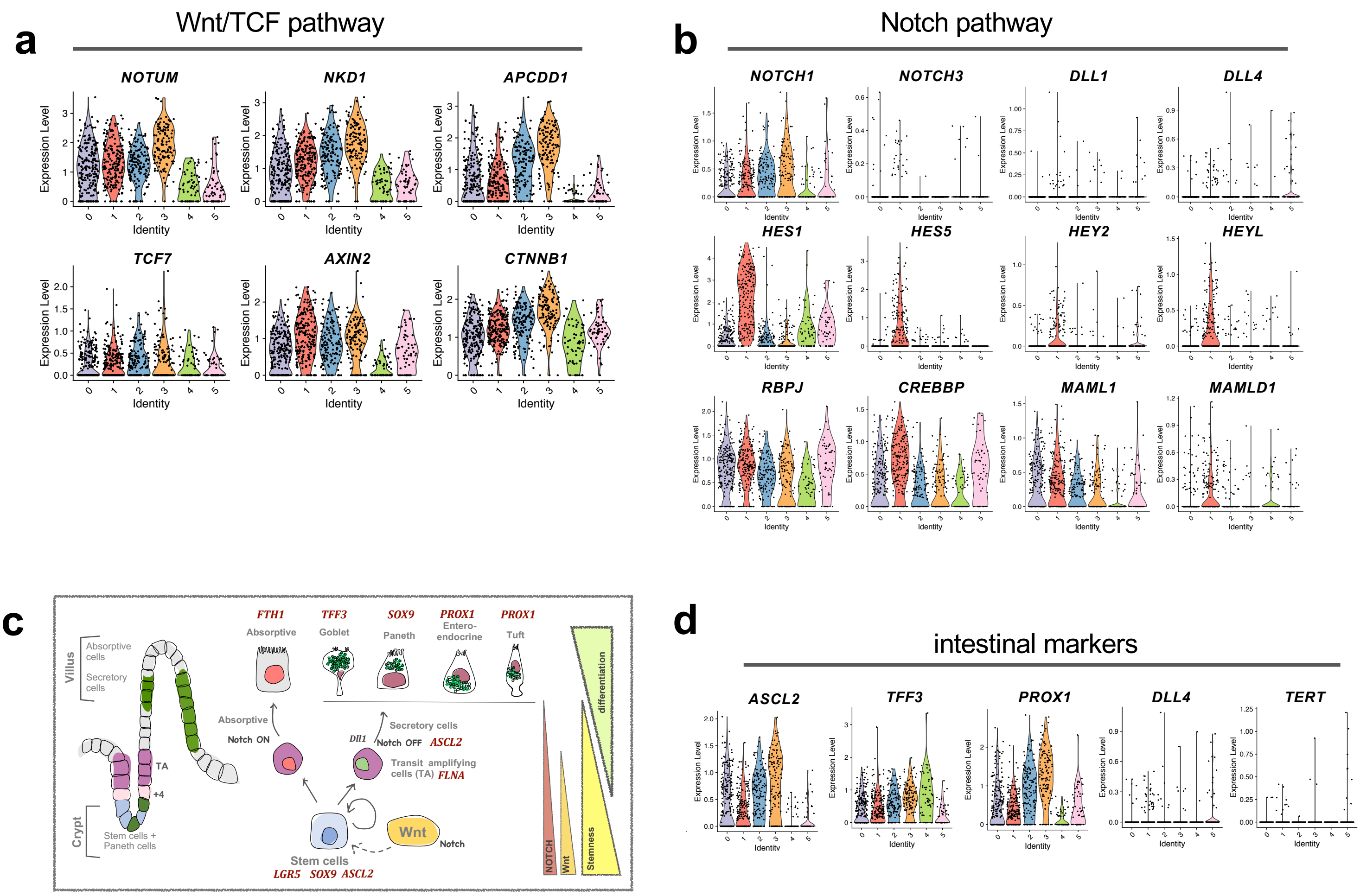

Supplementary Figure 10 | Gene expression profile of Wnt and Notch pathways and intestine cell lineage markers. **a-b.** Violin-plot of normalized expression values of representative genes belonging to the Wnt/Tcf (**a**) and Notch pathways (**b**). **c**. Schematic diagram of the different cell populations and simplified differentiation of the intestinal populations and some of the representative cell-fate lineages markers. **d**. Violin-plot of normalized expression values of representative *ASCL2, TFF3*, *PROX1*, *DLL4* and *TERT*. Each dot in the violin plot represents a different barcode (cell). Cells are divided according to their cellular subgroup.
