# Supplementary material for: Innate immune evasion revealed in a colorectal zebrafish xenograft model
Source: Nat Commun. 2021 Feb 19;12:1156. doi: 10.1038/s41467-021-21421-y (PMC7895829; doi:10.1038/s41467-021-21421-y)
Supplement: Supplementary file 3 — Descriptions of Additional Supplementary Files [file 41467_2021_21421_MOESM3_ESM.pdf]

Descriptions of Additional Supplementary Files

Supplementary Data 1

Description: 459 DEGs in SW480 vs SW620 comparison with a cutoff of FDR1-related to Figure 1d.

Supplementary Data 2

Description: Table with GSE-NES values of selected pathways related to Figure 8d.

Supplementary Software 1

Description: Code used in the analysis of the sequencing data presented in the manuscript. Respectively, File R_code1.R for bulk RNAseq analysis; File R_code2.R for single cell RNAseq analysis.
