# Supplementary material for: Innate immune evasion revealed in a colorectal zebrafish xenograft model
Source: Nat Commun. 2021 Feb 19;12:1156. doi: 10.1038/s41467-021-21421-y (PMC7895829; doi:10.1038/s41467-021-21421-y)
Supplement: Supplementary file 7 — Reporting Summary [file 41467_2021_21421_MOESM7_ESM.pdf]

nature research

Corresponding author(s): Rita Fior

Last updated by author(s): 16/01/21

Reporting Summary

Nature Research wishes to improve the reproducibility of the work that we publish. This form provides structure for consistency and transparency in reporting. For further information on Nature Research policies, see Authors & Referees and the Editorial Policy Checklist .

Statistics

For all statistical analyses, confirm that the following items are present in the figure legend, table legend, main text, or Methods section.

n/a	Confirmed	
☐	☒	The exact sample size (*n*) for each experimental group/condition, given as a discrete number and unit of measurement
☐	☒	A statement on whether measurements were taken from distinct samples or whether the same sample was measured repeatedly
☐	☒	The statistical test(s) used AND whether they are one- or two-sided<br>*Only common tests should be described solely by name; describe more complex techniques in the Methods section.*
☒	☐	A description of all covariates tested
☐	☒	A description of any assumptions or corrections, such as tests of normality and adjustment for multiple comparisons
☐	☒	A full description of the statistical parameters including central tendency (e.g. means) or other basic estimates (e.g. regression coefficient) AND variation (e.g. standard deviation) or associated estimates of uncertainty (e.g. confidence intervals)
☐	☒	For null hypothesis testing, the test statistic (e.g. *F*, *t*, *r*) with confidence intervals, effect sizes, degrees of freedom and *P* value noted<br>*Give P values as exact values whenever suitable.*
☒	☐	For Bayesian analysis, information on the choice of priors and Markov chain Monte Carlo settings
☒	☐	For hierarchical and complex designs, identification of the appropriate level for tests and full reporting of outcomes
☐	☒	Estimates of effect sizes (e.g. Cohen's *d*, Pearson's *r*), indicating how they were calculated

Our web collection on statistics for biologists contains articles on many of the points above.

Software and code

Policy information about availability of computer code

Data collection	Zeiss Zen software from Zeiss confocal microscope (Zen blue edition) and FIJI/Image J software, FlowJoTM v10.6.1 software for Flow Cytometry analysis, FACS Diva v8 for sorting.<br>For RNA seq, NextSeq500 Illumina sequencer was used.
Data analysis	SW480 expressing GFP and Sw620 expressing TdTomato were sorted in BD FACSAria Fusion with 99% of purity, usingFACS Diva software v8.<br><br>GraphPad Prism 8.0 software and Excell.<br><br>For RNAseq data: mRNA-libraries were prepared using the Smart-seq2 protocol (Illumina, USA). Samples were sequenced by Next-Seq 500 Illumina sequencer and unstranded single-end mRNA-seq libraries of 76bp were obtained. An average of approximately 38 millions of reads per sample. These RNA-seq libraries contain a mixture of human and zebrafish RNA derived from the xenograft as well as the host cells infiltrating it. After quality control assessment with FastQC (v0.11.7) and low quality reads filtering with Trimmomatic (v0.38), all sequenced libraries were quantified with Salmon (v0.13.1 using the respective transcript human annotations (Hg38) from the Ensembl genome database project). For downstream analysis package Tximport was used, to import transcript lengths and abundance estimates and export (estimated) count matrices. And differential expression analysis was performed using Limma. Genes with a FDR <0.05 and absolute log2 foldchange >1 were considered significant.<br>Pathway Enrichment Analysis of a ranked gene list using GSEA: Pathway enrichment analysis helps us gain biological insight into large gene lists typically resulting from high throughput experiments. It identifies biological pathways that are enriched in the gene list more than expected by chance. A ranked gene list obtained from SW480 low engraftment versus SW620 high engraftment differential expression analysis was input to GSEA PreRank (v4.0.2, Broad Institute, Cambridge, MA) as RNK file. We used curated gene sets from Molecular Signatures Database (v7.0, Hallmarks and Canonical Pathways including KEGG and REACTOME). We then ran GSEA PreRank using the default weighted statistic. The thresholds for significance were determined by permutation analysis (1000 permutations), selecting the enriched pathways with a false discovery rate (FDR) of < 0.07.<br>Single cell RNAseq analysis: During sequencing, Read 1 was assigned 26 base pairs and was used for identification of the Illumina library

barcode, cell barcode and Unique Molecular Identifiers (UMI). R2 was assigned 60 base pairs and used to map to the Human reference transcriptome with BWA74.Unique barcode gene counts were used for further processing in Seurat39. Namely, only genes present in at least 10 cells were considered. Moreover, we only considered barcodes with counts in more than 2000 genes and with less than 25% of counts in mitochondrial genes. After quality control and filtering we were left with a total of 533 human GFP positive cells from the first time-point and 293 human GFP positive cells from the second. After normalization, the 2000 most variable genes were used for dimensionality reduction and clustering. We chose clustering parameters empirically to provide a balance between the number of clusters and their size. We then ran GSEA analysis for each cluster by ranking genes according to their differences in gene expression to the other clusters. Cells were displayed in a 2-dimensional plot using uniform manifold approximation and projection (UMAP)40. Normalized expression values of genes from selected enriched pathways were also displayed as violin plots or heatmaps.

Data from the bulk and single-cell RNA-seq analyses have been deposited in NCBI Gene Expression Omnibus (GEO) with the accession number GSE163751 (https://www.ncbi.nlm.nih.gov/geo/query/acc.cgi?acc=GSE163751).

For manuscripts utilizing custom algorithms or software that are central to the research but not yet described in published literature, software must be made available to editors/reviewers. We strongly encourage code deposition in a community repository (e.g. GitHub). See the Nature Research guidelines for submitting code & software for further information.

Data

Policy information about availability of data

All manuscripts must include a data availability statement. This statement should provide the following information, where applicable:

- Accession codes, unique identifiers, or web links for publicly available datasets
- A list of figures that have associated raw data
- A description of any restrictions on data availability

The data that support the findings of this study are available from the corresponding author upon reasonable request.
RNA data deposit
Data from the bulk and single-cell RNA-seq analyses have been deposited in NCBI Gene Expression Omnibus (GEO) with the accession number GSE163751 (https://www.ncbi.nlm.nih.gov/geo/query/acc.cgi?acc=GSE163751).
We also provide an EXCELL with the raw data of each figure

Field-specific reporting

Please select the one below that is the best fit for your research. If you are not sure, read the appropriate sections before making your selection.

- [x] Life sciences
- [ ] Behavioural & social sciences
- [ ] Ecological, evolutionary & environmental sciences

For a reference copy of the document with all sections, see nature.com/documents/nr-reporting-summary-flat.pdf

Life sciences study design

All studies must disclose on these points even when the disclosure is negative.

Sample size

No sample-size calculation was performed.
In the zAvatars-Fig 1b- the limiting factor is the size of the sample (number of patient-derived tumor cells we can dissociate and obtain) and then the survival of the zAvatars- i.e. we do as much as we can. And the numbers are what we get.
In all other zebrafish xenografts experiments using cell lines we prepared always tumor cells in excess and tried to inject as much as possible zf embryos, limiting factor is the nº of the embryos that were layed and then the xenografts that died or acquired cardiac edema. Also sometimes the limiting is the penetrance of the transgenes (mpeg/tnf)– that in some cases gives low positive transgenics. We found empirically that with a minimal of 6-7 xenografts we can do the stats, but always aimed at having ~or more 15 xenografts analyzed per condition.
When analyzing engraftment we found that we need to have higher numbers for the Fisher exact test- we found we needed to have experiments with >30 xenografts to be more confident on our results. The experiments where we show more data is in the engraftment scoring because of this and also because is the gathering of several years of experiments performed by different students.

To quantify engraftment, tumor size, mitotic figures, apoptosis, % of neutrophils and macrophages we analyzed confocal images acquired in the Zeiss LSM 710. The minimum number of zebrafish larvae analyzed per experiment was 6 and maximum was 1408.

Data exclusions

We excluded 3 values - statistical outliers, determined by GraphPad QuickCalcs: outlier calculator, one in SW480 Fig3d and another Fig3i - values above 90% of neutrophils and macrophages in the SW480 TME. The last in Supplementary Figure 3 - SW480 tumor against macrophages.

Replication

To verify the reproducibility of the experimental findings most experiments were repeated at least three times. The exception being: zAvatars (Fig. 1b), that was only done once due to the limited amount of human sample; quantification of macrophages in zEscapers (Fig. 7h) and

phagocytosis (Supplementary Figure 4e); the mice experiments which was only performed once; % TAMS in MIX tumors (Fig.4g, N=12) and quantification of macrophages and neutrophils upon clodro treatment (Supplementary Figure 6).

We confirm that all attempts at replication were successful - with exception of SW480 engraftment that showed a very high variability (high coefficient variation) as we show in Fig1a- i.e. experiments presented a low engraftment rate- many times was zero- and we could not use these experiments – no xenografts were left to analyze. But some other times engraftment was >40% - which was rare but it did happen.
After analyzing the scRNA data we believe that maybe these cells have a high variability due to the cell-cell signaling pathways that are ON and therefore lead to fluctuations of gene expression that can impact on the phenotype.
Also, we are confident on our results because we observed that independently of the absolute value of engraftment for SW480 – panther mutants showed ~double that engraftment. For example if engraftment of SW480 was ~10% , then in Panther mutants we got ~20%, but if SW480 was ~30% in panther we observed ~60%, i.e. the relative phenotypes were always confirmed. The same happened when we do the MIX with SW620 (mix always ~double of the SW480 independent of the absolute value).

The number of independent experiments realized for each cell line is indicated in the corresponding image/subtitle.

Randomization

Before injection, we randomly mix a pool of zebrafish larvae and anesthetize them, in the moment of injection we randomly take some larvae for injection. 1day post injection we sort xenografts according to their tumor size and then randomly distributed in each experimental group. Mice experiments mice were randomized to be injected and were performed by experienced technician that randomly injected mice with the tumor cells.

Blinding

The experimental work was done in their majority by Vanda Povoa with exception of mice experiments and RNAseq analysis. Vanda and Catia performed zebrafish quantifications - given the fact that each cell line presents a very distinctive phenotype it is not possible be blind at all.
Micaela Domingues, Daniel Sobral and Ana Rita Grosso performed bioinformatic analysis. Mauro Muraro from Single Cell Discoveries performed the first data scRNAseq analysis in a completely blind way (no info on the project and expected results). Mayra Martinez-Lopez and Carlos Silva performed mice experiments and measured he mice xenografts tumors in a blind way since the mice ID are very complicated and mice were randomly distributed in the cages. Mayra Martinez-Lopez performed flow cytometry analysis.
Mauro Muraro from Single Cell Discoveries performed the first data analysis from scRnaseq in a blind way - out of the project.

Reporting for specific materials, systems and methods

We require information from authors about some types of materials, experimental systems and methods used in many studies. Here, indicate whether each material, system or method listed is relevant to your study. If you are not sure if a list item applies to your research, read the appropriate section before selecting a response.

Materials & experimental systems

n/a	Involved in the study	
☐	☒	Antibodies
☐	☒	Eukaryotic cell lines
☒	☐	Palaeontology
☐	☒	Animals and other organisms
☐	☒	Human research participants
☒	☐	Clinical data

Methods

n/a	Involved in the study	
☒	☐	ChIP-seq
☐	☒	Flow cytometry
☒	☐	MRI-based neuroimaging

Antibodies

Antibodies used

For immunofluorescence:
Primary antibodies for immuno fluorescence
-Anti-Cleaved Caspase-3 (rabbit, CST#9661, Asp175),
-Mpx (rabbit, GeneTex, 1:50, #gtx128379)
secondary antibodies:
- goat Anti-Rabbit IgG (H+L),
-DyLight 650 conjugated)

For flow cytometric analysis-monoclonal antibodies:
-Live/dead discrimination (LIVE/DEAD® Fixable Aqua Dead Cell Stain Kit, Thermo Scientific),
-anti-human EpCAM CD326 (clone 9C4, Lot B182124, Biolegend),
-APC/Cyanine7 anti-mouse CD45.2 (Clone 104, Lot: B240422, Biolegend),
-anti-mouse F4/80 PE-Cyanine7 (Clone BM8, Lot: 4281123, eBioscience),
-FITC anti-mouse CD80 (Clone 16-10A1, Lot: B207221, Biolegend).

Validation

Validation data for Cleaved Caspase-3 (Cell Signaling Technology, #9661) is available on the homepage of the manufacterer.
For Mpx antibody we performed immuno staining in transgenic zebrafish embryos that express GFP under the mpx promoter and observed a very good co-localization by confocal microscopy. This is the only antibody that we re-validated ourselves in our Lab since all the others were validated by the manufacturers and multiple publications available in the manufacturer website.

Validation data for Flow Cytometry antibodies is available on the homepage of the manufacterer Biolegend, eBiosceince and Thermo Scientific.

Eukaryotic cell lines

Policy information about cell lines

Cell line source(s)	Human breast cancer cell lines Hs578T, MDA-MB-231 and MDA-MB-468 were derived from American Type Culture Collection and kindly provided by Monica Bettencourt Dias'Lab (Instituto Gulbenkian de Ciência). Human colorectal cancer cell lines SW480, SW620 and HT29 were purchased from ATCC, whereas HCT116 and Hke3 isogenic cell lines were derived from American Type Culture Collection and kindly provided by Ângela Relógio (Charité Medical University of Berlin). SW48 colorectal cancer cell line was provided by Luis Costa Lab (ATCC, Instituto de Medicina Molecular).
Authentication	All cell lines were authenthicated through short tandem repeat (STR) profile analysis.
Mycoplasma contamination	All cell lines were tested and were negative for mycoplasma.
Commonly misidentified lines (See ICLAC register)	The study did not involve misidentified lines.

Animals and other organisms

Policy information about studies involving animals; ARRIVE guidelines recommended for reporting animal research

Laboratory animals	In vivo experiments were performed in zebrafish model (Danio rerio), which was maintained and handled in accordance with European Animal Welfare Legislation, Directive 2010/63/EU and Champalimaud Fish Platform Program.<br>Adult zebrafish were kept in 3.5L tanks with a maximum population of 30 fish per tank, both male and female, in a running water system, feeded twice a day and maintained in a temperature and humidity controlled environment, as well as, day-night automatic cycle of 14 hours light plus 10 hours dark.<br>Adults were used to breed and the experiments were performed in 2dpf zebrafish larvae.<br>According to the purpose of each experiment, different genetically modified zebrafish lines were used in this study: Tg (mpx:eGFP), Tg(mpeg1:mCherry-F), Tg(mpeg1:mCherry-F;tnfa:GFP-F), runx1 w84x mutant, csfr1a j4blue panther mutant. Wild-type Tubingen or Casper mutants were used as control and as a background line for experiments.<br><br>Rag1-/- C57BL/6J mice were bred at 23°C, with 40-60% relative humidity, 12hrs light cycle (8am-8pm) by the animal facility of Champalimaud Vivarium, Lisbon, Portugal.<br>For SW480, SW620 and MIX mice xenografts 7~10 weeks old Rag1-/- C57BL/6J mice were used (SW480-GFP N=5, 2F+3M, SW620-Tomato N=5, 2F+3M and MIX N=5, 2F+3M).<br>For L-Clodronate experiment 8 weeks old Rag1-/- C57BL/6J mice were used (N=6 per group - PBS: 3F+3M; L-PBS: 3F+3M; L-Clodro: 4F+2M). One F of PBS condition and one F of L-Clodro condition died during the experiment.
Wild animals	The study did not involve wild animals.
Field-collected samples	The study did not involve samples collected from the field.
Ethics oversight	All animal experiments and protocols (Zebrafish and Mice) were approved by the Champalimaud Animal Ethical Committee and Portuguese institutional organizations- ORBEA (Órgão de Bem-Estar e Ética Animal / Animal Welfare and Ethics Body) and DGAV (Direção Geral de Alimentação e Veterinária / Directorate General for Food and Veterinary).

Note that full information on the approval of the study protocol must also be provided in the manuscript.

Human research participants

Policy information about studies involving human research participants

Population characteristics	N/A we had access to very small number of patients so we could not discriminate in different groups.
Recruitment	The project was explained to patients from Centro Clínico Champalimaud and Hospital Professor Fernando da Fonseca and patients become as participants after signing an informed consent if interested in joining the study. There was no bias in selecting patients- the surgeons are the ones that enroll the patients in the study according to clinical criteria.
Ethics oversight	Human samples used for zebrafish patient-derived xenograft (zAvatars) establishment were obtained from Champalimaud Hospital and Prof Fernando Fonseca Hospital with written informed consent. The study was approved by both Hospital Ethics Committees.

Note that full information on the approval of the study protocol must also be provided in the manuscript.

Flow Cytometry

Plots

Confirm that:

- [x] The axis labels state the marker and fluorochrome used (e.g. CD4-FITC).
- [x] The axis scales are clearly visible. Include numbers along axes only for bottom left plot of group (a 'group' is an analysis of identical markers).
- [x] All plots are contour plots with outliers or pseudocolor plots.
- [x] A numerical value for number of cells or percentage (with statistics) is provided.

Methodology

Sample preparation	Tumor-bearing mice were euthanized according to approved guidelines with carbon dioxide three weeks after inoculation of cancer cells. Subcutaneous tumors were resected and measured with a caliper. Tumors were thoroughly minced with scalpels and then transferred to 1,5mL Eppendorf tubes and digested in PBS 1X containing Liberase TM (Sigma) and DNAse I (Thermo Scientific) for 30min at 37ºC. Digested suspension was filtered through a 40um mesh into a 15 mL Falcon tube. Digestion was then blocked by addition of buffer containing HBSS (Corning), EDTA and BSA. Tubes were centrifuged 10min at 300g. Pellet was resuspended in FACS buffer. Total viable cell yield per volume was determined using Trypan Blue and an automated cell counter. Tumor single cell suspension was then stained for FACS analysis.
Instrument	BD LSRFortessa X-20 cell analyzer (Biosciences) was used for data collection
Software	FlowJoTM v10.6.1 software for Flow Cytometry analysis, FACS Diva v8 for sorting.
Cell population abundance	~4x10^6 SW480 expressing GFP and Sw620 expressing TdTomato after lentivirus transduction were sorted in BD FACSAria Fusion with 99% of purity, usingFACS Diva software v8, and then cryopreserved for future usage.
Gating strategy	Data was acquired using the BD LSRFortessa™ X-20 cell analyzer (Biosciences) and analyzed using FlowJo™ v10.6.1 software. Populations were determined as follows: Human cells (LIVE/DEAD-EpCAM+), SW620 cells (LIVE/DEAD-EpCAM+PE+FITC-), SW480 cells (LIVE/DEAD-EpCAM+PE-FITC+), Mouse cells (LIVE/DEAD-EpCAM-), Macrophages (LIVE/DEAD-EpCAM-CD45+F4/80+), anti-tumoral M1-like macrophages (LIVE/DEAD-EpCAM-CD45+F4/80+CD80+CD163-), pro-tumoral M2-like macrophages (LIVE/DEAD-EpCAM-CD45+F4/80+CD80-CD163+).

- [x] Tick this box to confirm that a figure exemplifying the gating strategy is provided in the Supplementary Information.
